# Navigating the Genetic Frontier for the Integration of Genetic Services into African Healthcare Systems: A scoping review

**DOI:** 10.21203/rs.3.rs-3978686/v1

**Published:** 2024-02-28

**Authors:** Karen Kengne Kamga, Marlyse Pallissa Fonkam, Séraphin Nguefack, Ambroise Wonkam

**Affiliations:** University of Cape Town; Regional Hospital Limbe; University of Yaoundé I; University of Cape Town

**Keywords:** Genetic Services, Africa, Healthcare, Challenges, Opportunities, Community Engagement

## Abstract

**Background:**

The integration of genetic services into African healthcare systems is a multifaceted endeavor marked by both obstacles and prospects. This study aims to furnish evidence-based recommendations for policymakers and healthcare entities to facilitate the effective assimilation of genetic services within African healthcare systems.

**Methods:**

Employing a scoping review methodology, we scrutinized peer-reviewed studies spanning from 2003 to 2023, sourced from PubMed, Scopus, and Africa-wide databases. Our analysis drew upon eight pertinent research studies conducted between 2016 and 2023, encompassing diverse genetic topics across six African nations, namely Cameroon, Kenya, Nigeria, Rwanda, South Africa, and Tanzania.

**Results:**

The reviewed studies underscored numerous challenges hindering the implementation of genetic services in African healthcare systems. These obstacles encompassed deficiencies in disease awareness and education, impediments to genetic testing, resource scarcities, ethical quandaries, and issues related to follow-up and retention. Nevertheless, the authors also identified opportunities and strategies conducive to successful integration, emphasizing proactive measures such as community engagement, advocacy, and the fostering of supportive networks.

**Conclusion:**

The integration of genetic services in Africa holds promise for enhancing healthcare outcomes but also poses challenges and opportunities for healthcare and biotechnology enterprises. To address gaps in disease awareness, we advocate for healthcare providers to invest in educational initiatives, forge partnerships with local institutions, and leverage digital platforms. Furthermore, we urge businesses to innovate and devise cost-effective genetic testing models while establishing online forums to promote dialogue and contribute positively to African healthcare.

## 1 Background

Genetic services (GSs) that have revolutionized healthcare include genetic testing (GT), genetic counselling (GC), and personalized medicine (PM) [1, 2]. Genetic testing is used to analyse an individual’s DNA to identify any genetic variations associated with certain health conditions [3], while genetic counselling involves educating individuals and families about genetic risks, inheritance patterns, and the implications of genetic test results [4]. Personalized medicine utilizes genetic information to tailor treatment plans, identify disease risk factors, and offer preventative measures that are specific to an individual’s genetic profile [5]. By leveraging the power of genetic services, healthcare providers can offer a more comprehensive and personalized approach to patient care, leading to better health outcomes.

The integration of genetic services in African healthcare systems is a crucial step toward improving the overall quality of healthcare on the continent [6]. Genetics plays a significant role in understanding and treating various diseases, and by incorporating genetic services into healthcare systems, African countries can enhance their ability to provide accurate diagnoses and personalized treatment plans [6, 7].

One of the main benefits of integrating genetic services is the early detection and prevention of genetic disorders [8, 9]. By identifying individuals who are at risk for certain genetic conditions, healthcare providers can offer appropriate counselling and interventions to reduce the impact of these disorders, as described by Rubin et al. (2005), who described healthcare workers improving adherence to treatment in health facilities through counselling [10]. This proactive approach can significantly improve patient outcomes and reduce the burden on healthcare systems in the long run.

Furthermore, incorporating genetics into African healthcare systems can also contribute to advancements in medical research [11, 12]. Genetic data collected from patients can be used for scientific studies that aim to understand disease mechanisms [13, 14], develop new treatments [15, 16], and identify potential targets for drug development [17]. This research has the potential to not only benefit Africans but also contribute to global medical knowledge.

A 2015 systematic review by Mikat-Stevens and colleagues examined the obstacles and enablers associated with using genetic services among primary care providers. Their analysis revealed that the majority of the studies evaluated were conducted in Europe and the United States, with limited representation of African literature [18]. While their findings highlighted resource constraints, insufficient personnel training, and ethical considerations related to privacy and consent, applying these results to the African context can be difficult due to the diverse nature of the population and their cultures [19].

The integration of genetic services in African healthcare systems poses both opportunities and challenges. On the one hand, it holds great promise for revolutionizing healthcare by offering personalized and targeted treatments for genetic disorders [20]. However, it also raises concerns about accessibility, affordability, and cultural acceptance. Investment in infrastructure, training programs, and awareness campaigns are needed [21]. By addressing these issues head-on through international collaboration and technological advancements, Africa can utilize the power of genetics to improve healthcare outcomes for its population.

This study aims to offer evidence-based suggestions that can aid policymakers and healthcare organizations in enhancing the integration of medical genetic services in Africa. We formulated a research question to identify the current challenges and strategies for integrating genetic services within African healthcare systems. By answering this question, we hope to contribute to the advancement of healthcare services in Africa and assist in addressing the unique challenges faced by the region.

## 2 Methodology

### 2.1 Research Design:

I will conduct a scoping review, which aimed to gather a comprehensive range of information by including qualitative and quantitative studies alongside case studies and interviews with key stakeholders in the field from African scholars. The timeframe spans from 2003 to 2023.

### 2.2 Search strategy

The process of conducting a thorough review of the integration of genetic services within African healthcare systems is multifaceted and intricate and involves several crucial steps [22]. To ensure a comprehensive and exhaustive review, I used three of the most popular databases available, namely, PubMed, Scopus, and African Wide Information, in my initial search. I also used appropriate and relevant keywords derived from “genetic services” and “Africa” to conduct my searches, as outlined in Table 1. This guaranteed that a broad range of literature, including research articles, systematic reviews, and meta-analyses, were captured and that no relevant studies were overlooked.

To ensure that I captured the most recent developments in the field of genetic services, I included only studies published in English from the period spanning 2003 to 2023. In addition to peer-reviewed literature, I also scoured gray literature, conference proceedings, and relevant reports to ensure that my review was comprehensive and exhaustive. By conducting this thorough review, I hope to better understand the current state of genetic service integration within African healthcare systems and identify areas that require further research and improvement.

### 2.3 Study selection

**Figure 1:**
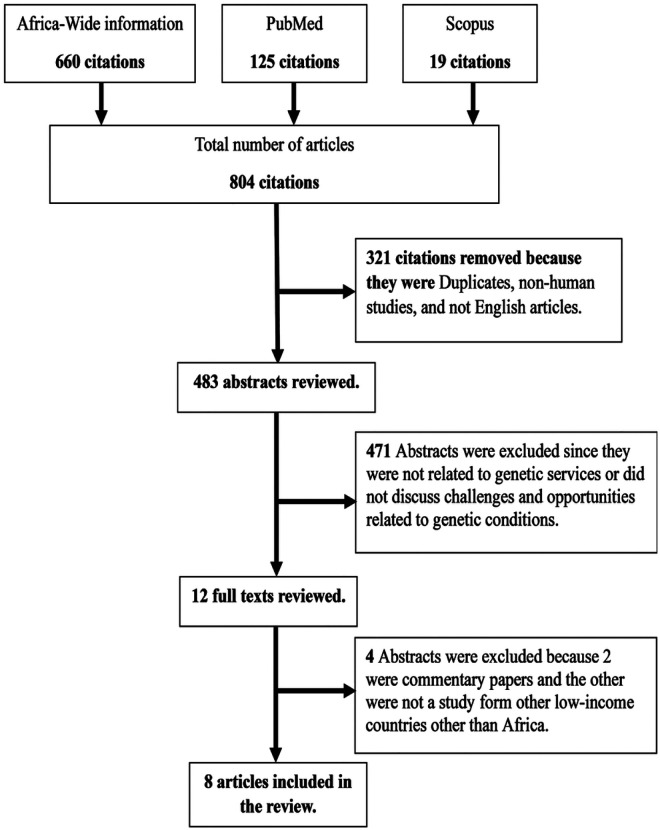
Study selection process

To ensure the efficiency and accuracy of my review process, it is crucial to establish well-defined inclusion and exclusion criteria. My focus was to gather information on the integration of genetic services within African healthcare systems through primary research. Therefore, only peer-reviewed studies that directly address my research question were included in my review. To provide a comprehensive understanding of the topic, I excluded those unrelated to genetic services within the African context. It is important to note that my inclusion criteria were limited to studies published in English between 2003 and 2023. My review process followed a rigorous quality assessment procedure, and I included only studies that met my predefined quality standards (Fig. 1). The search was conducted on the 1st of November 2023.

### 2.4 Data extraction and analysis

During the data extraction and analysis phase, it is crucial to establish a comprehensive data extraction form that can efficiently capture all the relevant information from the included studies [23]. The form aimed to capture the essential study characteristics, methodologies, key findings, challenges, opportunities, and strategies. To perform my data analysis, I used NVIVO 14, which enabled me to use the framework analysis to thoroughly understand my data and provide me with a narrative synthesis of my findings based on the identified themes and subthemes.

Moreover, to ensure the quality and rigour of the included studies, I conducted a third-party review of the reviewed studies and the review process. This third party will scrutinize the theme’s notations and provide their opinions on the discussion of any areas of disagreement. The ultimate goal was to reach a consensus on whether the article should be obtained and included in the review.

## 3 Results

### 3.1 Description of the study strategy:

We conducted a thorough search and found eight studies on genetics in six African countries between 2016 and 2023. These studies explored different aspects of genetic counselling and testing, primarily focusing on addressing challenges and possible opportunities (Table 2). In Cameroon, two studies conducted in 2021 and 2023 examined challenges and solutions in genetic counselling for hearing impairment and cancer, respectively. A study in Kenya (2021) explored opportunities and barriers to genetic service delivery from a health personnel perspective. In Nigeria, a review conducted in 2017 focused on genetics and genomics, emphasizing prenatal diagnosis, while another study in 2023 delved into sickle cell disease with an emphasis on premarital genetic counselling. Rwanda stressed the importance of genetic testing for breast cancer through a commentary published in 2016. A study conducted in South Africa in 2023 reviewed schizophrenia, addressing genetic counselling challenges, and Tanzania reported a quantitative study on sickle cell disease in 2022, emphasizing neonatal diagnosis and follow-up.

### 3.2 Themes identified.

Table 3 summarizes the key challenges and opportunities that arise during the implementation of genetic services. To effectively implement genetic services, it is important to identify specific themes and subthemes that can address challenges and maximize opportunities.

One of the main challenges in implementing genetic services is the need for more awareness and education regarding genetic diseases. It is also important to identify and resolve barriers that hinder the genetic testing process, making it more accessible and efficient. Resource limitations and restricted access to genetic services are also significant challenges that need to be overcome for widespread access. Ethical considerations in genetic services require addressing ethical dilemmas associated with genetic testing and counselling.

On a positive note, we have identified some opportunities for the effective implementation of genetic services. Supportive networks and advocacy for genetic services are essential opportunities that can help promote awareness and accessibility. Community engagement is also crucial for involving communities for better acceptance and understanding.

## 4 Discussion

In our study, the challenges confronting the implementation of genetic services in Africa were extensively documented. Hamilton et al. (2014) highlighted the imperative for a comprehensive and multifaceted approach to surmount the impediments obstructing the effective establishment of genetic services throughout the continent [32]. Among the primary challenges identified are the imperative need for disease awareness within the population, a challenge rooted in various factors such as limited access to information, inadequate healthcare infrastructure, and pervasive cultural stigmas surrounding genetic disorders. Chapple et al. (1995) also reported that limited access to information is one of the challenges to accessing genetic services [33]. Establishing a foundational understanding of the significance of genetic services is pivotal, as it can potentially lead to increased community engagement, thereby advancing genetic healthcare in Africa.

Our study also elucidates the scarcity of practical expertise and infrastructure essential for genetic services in Africa, emphasizing the criticality of building a skilled workforce and establishing robust infrastructure for the success of genetic services. Hooker (2021) articulated the importance of building an infrastructure to enable the delivery of genomic medicine [34]. The exorbitant costs associated with genetic testing and counselling services present another major obstacle, particularly in resource-limited settings, where financial constraints can significantly impede access. Furthermore, the unavailability of essential equipment compounds the challenges faced in implementing genetic services, underscoring the pressing need for financial investments and collaborative efforts to fortify capacity and enhance the infrastructure for genetic healthcare across the African continent.

In addition to these challenges, ethical dilemmas further complicate the implementation of genetic services in Africa, with legal constraints on selective abortion being a prominent concern that may restrict choices available to individuals and families following the identification of genetic disorders. Blackshaw (2020) argued that a liberal pro-choice stance on selective abortion for disability implies the permissibility of selective abortion for a wide range of genetic traits [35]. Language barriers also contribute to ethical challenges by hindering effective communication between healthcare providers and patients, highlighting the need for nuanced strategies that navigate cultural sensitivities, legal frameworks, and the diverse linguistic landscape across the continent [36].

Despite these challenges, this study identified several opportunities and strategies that could contribute to the successful implementation of genetic services in Africa. Prevention through community engagement and education emerges as a pivotal strategy, advocating for increased awareness about the importance of genetic services and dispelling prevalent myths and misconceptions, empowering communities to become proactive participants in their healthcare [37]. Supportive networks play a vital role in offering emotional and informational assistance to individuals and families navigating the complexities of genetic testing and counselling. Rantanen et al. (2008) discovered that although the ideal of genetic counselling remains consistent across guidelines, contradictions arise between the need for objective information and the counsellor’s adaptation to the circumstances of the counselee [38]. Additionally, multidisciplinary approaches involving collaboration between healthcare professionals, researchers, and community leaders can contribute to a more holistic and culturally sensitive implementation of genetic services [39].

Another promising strategy is establishing community advisory boards, which act as intermediaries between healthcare providers and the community to ensure that genetic services are tailored to meet diverse populations’ specific needs and concerns. A recent study conducted by O’Daniel et al. (2022) revealed that language barriers in healthcare can have serious consequences, such as miscommunication, reduced satisfaction, and a lower quality of healthcare delivery, ultimately leading to compromised patient safety. However, the study suggested that having an intermediary between healthcare providers and patients can facilitate communication and improve satisfaction among both parties. In particular, a community advisory board concerning genetic conditions, which serves as an intermediary, can enhance healthcare quality and promote patient safety [40]. Moreover, advocacy efforts are pivotal in driving policy changes and securing resources for genetic healthcare. Martinez et al. (2020) emphasized that developing national registries for patients with SCD is of utmost importance. They also highlighted the crucial role that patient advocacy and community engagement groups play in achieving this goal [41]. An inclusive and sustainable approach to genetic services in Africa must prioritize follow-up and retention strategies to maintain participant engagement. Addressing these opportunities and implementing comprehensive strategies holds the potential to overcome the challenges identified in the study and pave the way for effective and culturally sensitive genetic healthcare in Africa.

### 4.1 Implications

The implementation of genetic services in Africa presents challenges and opportunities that have significant business implications for healthcare, biotechnology, and genetic services companies. Understanding these implications is crucial for developing effective business strategies and contributing positively to the healthcare landscape in Africa.

Healthcare providers and scholars can play a pivotal role in addressing the lack of disease awareness and education by investing in educational programs targeting healthcare professionals and communities. This could involve collaboration with local institutions, the creation of informative materials, and the leveraging of digital platforms to disseminate educational content. Additionally, businesses can explore innovative and cost-effective models for genetic testing to overcome the barriers related to high costs and limited accessibility. This might involve developing affordable testing kits, partnering with local healthcare providers, or implementing telemedicine solutions to reach underserved populations. Furthermore, businesses can develop platforms facilitating dialogue between healthcare providers, genetic counsellors, and communities. This could involve the creation of online forums, mobile applications, or community outreach programs to disseminate information and address concerns.

### 4.2 Limitations

This scoping review has several limitations that might impact our findings. One of these limitations is the search strategy that we utilized, which concentrated solely on studies published in English. Other pertinent literature may have been published in different languages and may have been overlooked in this review. This could restrict the extent and thoroughness of our review and have an impact on the precision of its conclusions. As a result, it is essential to bear in mind this limitation while interpreting the outcomes of this review.

## 5 Conclusion

In conclusion, while challenges persist in implementing genetic services in Africa, the identified opportunities and strategies underscore the potential for positive change. Prioritizing prevention, leveraging supportive networks, engaging communities, and advocating for resource allocation are crucial components of a comprehensive approach to address the complexities of genetic services in the African context. These findings emphasize the need for tailored interventions that align with cultural values, involve diverse stakeholders, and prioritize education and community engagement to foster the successful integration of genetic services in Africa. Businesses must adopt a holistic and socially responsible approach to address the challenges and leverage the opportunities to implement genetic services in Africa. By aligning with the needs and values of local communities, businesses can contribute to advancing genetic services while building sustainable and impactful operations in the African healthcare landscape.

## Figures and Tables

**Table 1:** Search parameters and search terms for the scoping review

Search parameters	
Language	English
Period	2003–2023
Geographic population	Africa
Databases	PubMed, Scopus, African-wide information
Search terms	
Africa	Africa OR Algeria OR Angola OR Benin OR Botswana OR “Burkina Faso” OR Burundi OR Cameroon OR “Cape Verde” OR “Central African Republic” OR Chad OR Comoros OR Djibouti OR “DR Congo” OR “Democratic Republic of Congo” OR Congo OR Egypt OR “Equatorial Guinea” OR Guinea OR Eritrea OR Eswatini OR Ethiopia OR Gabon OR Gambia OR Ghana OR Guinea OR (Guinea Bissau) OR “Ivory Coast” OR “Cote d’Ivoire” OR Kenya OR Lesotho OR Liberia OR Libya OR Madagascar OR Malawi OR Mali OR Mauritania OR Mauritius OR Morocco OR Mozambique OR Namibia OR Niger OR Nigeria OR Rwanda OR “Sao Tome And Principe” OR Senegal OR Seychelles OR Sierra Leone OR Somalia OR “South Africa” OR “South Sudan” OR Sudan OR Tanzania OR Togo OR Tunisia OR Uganda OR Zambia OR Zimbabwe
Genetic services	(Pediatric genetic counselling) OR (newborn screening follow-up) AND (genetic* service* provision) OR (genetic* service* delivery) OR (genetic* service* delivery model*) OR (genetic health care service* delivery) OR (genetic* support delivery) OR (genetic* support model*) OR (genetic* service* delivery structure) OR (genetic* services delivery system) OR (genetic* delivery of health care) OR (genetic counselling services) OR (genetic diagnostic service*) OR (genetic counselling) OR (genetic* service*) OR (genetic* test*) OR (Personal* medicine) OR (Precision medicine)

**Table 2:** Description of the Key Research Articles

Title of article	First author	Affiliation of first author	country	year of publication	Study design	genetic condition	Genetic service	Summary of research
Anticipated ethical challenges with growing molecular prenatal diagnosis in Nigeria	Tahir [24]	Department of Medical Microbiology, Kaduna State University, Kaduna, Nigeria.	Nigeria	2017	Review	Molecular prenatal diagnosis	Prenatal diagnosis	Prenatal diagnostic testing (PDT) is increasingly popular in Nigeria, allowing mothers to detect genetic disorders in their unborn babies. However, ethical concerns arise regarding counselling, sample collection methods, and justification for the tests. There are also ethical dilemmas related to whether to consider abortion if the foetus is affected. This paper explores the ethical issues of genetic testing concerning the foetus, pregnant woman, society, counselling, and informed consent.
Barriers and Facilitators of Premarital Genetic Counselling for Sickle Cell Disease in Northern Nigeria	Galadanci [25]	Department of Hematology, Bayero University/Aminu Kano Teaching Hospital, Nigeria	Nigeria	2023	qualitative study	SCD	Premarital genetic counselling	Premarital genetic counselling for Sickle Cell Disease is not available in Nigeria. This study was conducted to understand the attitudes and beliefs towards SCD and premarital genetic counselling. Lack of knowledge, testing facilities, transportation, and stigma associated with the disease are barriers to premarital testing. However, there is a willingness within the community to receive premarital testing for SCD.
Breast Cancer in Africa ~ Limitations and Opportunities for Application of Genomic Medicine	Allison Silverstein [26]	Rwanda Military Hospital, Kigali, Rwanda	Rwanda	2016	commentary	Breast cancer	Genetic testing for cancer	Genomic medicine is not applied enough in low-income areas, causing health disparities. Breast cancer is a leading cause of cancer incidence and mortality in Africa, yet there is a lack of research into genetic determinants of the disease-specific to African populations. There is a need to strengthen research efforts and support new initiatives to integrate genomic medicine into breast cancer care in Africa. This will enable tailored treatment and maximum impact, even when resources are limited. Although there may be systemic barriers, implementing genomic medicine is essential to improve breast cancer outcomes and survival, especially in underserved populations.
Building research capacity for sickle cell disease in Africa ~ Lessons and challenges from establishing a birth cohort in Tanzania	Nkya [27]	Department of Haematology and Blood Transfusion, Muhimbili University of Health and Allied Sciences, Dar es Salaam, Tanzania	Tanzania	2022	quantitative prospective	SCD	Neonatal diagnosis and follow up	The establishment of SCD birth cohorts is a promising approach for early disease and intervention management. However, there are very few birth cohorts in Africa. In this research, we established a birth cohort of 341 babies with and without SCD and followed them for three years. Clinical and demographic information was collected during enrollment and follow-up visits that were synchronized with the immunization schedule. Through this study, we gained valuable insights into establishing and maintaining a research birth cohort in an African context. We learned the importance of integrating the cohort with the other existing programs to ensure its sustainability.
Ethical principles, challenges and opportunities when conducting genetic counselling for schizophrenia	Zingela [28]	Executive Dean’s Office, Nelson Mandela University, Port Elizabeth, South Africa	South Africa	2023	review	Schizoprenia	Genetic counselling	GC for schizophrenia presents ethical challenges that require attention. Clinicians and researchers must communicate complex scientific information in a way that patients and their families can easily understand. To achieve this, they should draw on lessons from South African studies and apply ethical principles such as beneficence, autonomy, informed consent, confidentiality, and distributive justice. The paper also emphasizes the importance of addressing multicultural and multilingual populations and establishing community advisory boards to handle ethical challenges. All parties involved should strive to share scientific information accurately, guided by empathy. Capacity and expertise in genetic counselling must be built through funding and resources.
Feasibility of cancer genetic counselling and screening in Cameroon ~ perceived benefits and barriers	Berthe Sabine Esson Mapoko [29]	FMBS university of yaounde 1	Cameroon	2023	quantitative	cancer	Genetic counselling and testing	This study at Yaoundé General Hospital, Cameroon, aimed to assess the acceptance, perceived benefits, barriers, and willingness to pay for genetic cancer screening among cancer patients in Cameroon. The study included cancer patients, of which 97.5% expressed their willingness to test for genetic mutations and were willing to pay for genetic testing. However, the study revealed barriers such as the cost, lack of equipment, and anticipated anxiety. The findings suggest that establishing a cancer risk assessment clinic for cancer in Cameroon could be helpful and guide the development of cancer screening programs.
Knowledge and Challenges Associated With Hearing Impairment in Affected Individuals From Cameroon (Sub-Saharan Africa)	edmond wonkam [30]	University of cape town	Cameroon	2021	qualitative study	hearing impaired	Genetic counselling	This study was conducted to identify challenges faced by people with hearing impairment in Cameroon and to suggest ways to improve their quality of life. Ten healthcare workers, educationists, and ten individuals with hearing impairment, including caregivers, were interviewed. The study revealed that people associate hearing impairment with genetics, environmental factors, and spiritual curses. It also found that individuals with hearing impairment have difficulties accessing education and healthcare. To address these issues, the government and health researchers should develop strategies for prevention and early diagnosis of hearing impairment, including awareness campaigns, facilities for proper management, newborn screening, and premarital screening to reduce the burden of genetic hearing impairment.
Opportunities and barriers for genetic service delivery in Kenya from a health personnel perspective	Adrina Zhong [31]	Department of Social and Behavioural Health Sciences, Dalla Lana School of Public Health, The University of Toronto, Toronto, ON, Canada	Kenya	2021	qualitative study	genetic counselling	Genetic counselling and testing	Like many developing countries, Kenya needs more licenced genetic counsellors and has limited access to genetic testing. This study identified opportunities and barriers to genetic service delivery in the Kenyan healthcare system by interviewing personnel who offer genetic services. The findings showed that education, training, costs, and counselling challenges were the main barriers, while demand, education, training, a multidisciplinary approach, and improved laboratory infrastructure were identified as opportunities.

**Table 3:** Description of the different themes identified – Challenges and Opportunities.

Main theme	Subtheme
Challenges of implementing genetic services	Lack of Disease Awareness and Education [24–26, 29–31]
	Barriers to Testing [24–26, 29, 31]
	Recourses Limitations and Access to Services [26–28, 31]
	Ethical Dilemmas [24, 28, 30]
	Follow-Up and Retention Challenges [27]
Opportunities to implementing genetic services	Prevention [24–26, 28, 29]
	Presence of supportive networks [26–28, 31]
	Advocacy [26, 31]
	Community engagement [26, 28]

## Data Availability

All data generated or analysed during this study are included in this published article in the references. They are all publicly available articles.
